# Investigating the Effect of COVID-19 on Driver Behavior and Road Safety: A Naturalistic Driving Study in Malaysia

**DOI:** 10.3390/ijerph191811224

**Published:** 2022-09-07

**Authors:** Ward Ahmed Al-Hussein, Wenshuang Li, Lip Yee Por, Chin Soon Ku, Wajdi Hamza Dawod Alredany, Thanakamon Leesri, Huda Hussein MohamadJawad

**Affiliations:** 1 Department of Computer System and Technology, Faculty of Computer Science and Information Technology, University of Malaya, Kuala Lumpur 50603, Malaysia; 2 Faculty of Business and Economics, University of Malaya, Kuala Lumpur 50603, Malaysia; 3 Department of Computer Science, Universiti Tunku Abdul Rahman, Kampar 31900, Malaysia; 4 Department of Mathematics, Dhofar University, Salalah 211, Oman; 5 School of Community Health Nursing, Institute of Nursing, Suranaree University of Technology, 111 University Ave., Muang, Nakhon Ratchasima 30000, Thailand; 6 College of Information Technology, Universiti Tenaga Nasional, Jalan IKRAM-UNITEN, Kajang 43000, Malaysia

**Keywords:** driving behavior, road safety, COVID-19, aggressive driving, naturalistic driving

## Abstract

The spread of the novel coronavirus COVID-19 resulted in unprecedented worldwide countermeasures such as lockdowns and suspensions of all retail, recreational, and religious activities for the majority of 2020. Nonetheless, no adequate scientific data have been provided thus far about the impact of COVID-19 on driving behavior and road safety, especially in Malaysia. This study examined the effect of COVID-19 on driving behavior using naturalistic driving data. This was accomplished by comparing the driving behaviors of the same drivers in three periods: before COVID-19 lockdown, during COVID-19 lockdown, and after COVID-19 lockdown. Thirty people were previously recruited in 2019 to drive an instrumental vehicle on a 25 km route while recording their driving data such as speed, acceleration, deceleration, distance to vehicle ahead, and steering. The data acquisition system incorporated various sensors such as an OBDII reader, a lidar, two ultrasonic sensors, an IMU, and a GPS. The same individuals were contacted again in 2020 to drive the same vehicle on the same route in order to capture their driving behavior during the COVID-19 lockdown. Participants were approached once again in 2022 to repeat the procedure in order to capture their driving behavior after the COVID-19 lockdown. Such valuable and trustworthy data enable the assessment of changes in driving behavior throughout the three time periods. Results showed that drivers committed more violations during the COVID-19 lockdown, with young drivers in particular being most affected by the traffic restrictions, driving significantly faster and performing more aggressive steering behaviors during the COVID-19 lockdown than any other time. Furthermore, the locations where the most speeding offenses were committed are highlighted in order to provide lawmakers with guidance on how to improve traffic safety in those areas, in addition to various recommendations on how to manage traffic during future lockdowns.

## 1. Introduction

Coronavirus disease (COVID-19) is an illness caused by the SARS-CoV-2 virus [1]. In 2019, the first confirmed COVID-19 cases were reported in Wuhan, Hubei Province, China [2]. Beginning in March of 2020, the World Health Organization (WHO) declared it a pandemic [3]. As of July 2022, the virus had infected more than 559 million individuals, with the death toll reaching 6.36 million. Malaysia was one of the countries severely affected by COVID-19. According to the Malaysian Ministry of Health, there were 4.6 million infections and 36,044 fatalities by 10 August 2022 [4].

The COVID-19 pandemic has caused significant disruptions in several businesses, with the transportation industry being among the most affected. Consequently, several researchers are attempting to comprehend the influence that COVID-19 has on road safety by investigating the dynamics of the pandemic in numerous countries [5].

According to studies across the globe, the COVID-19 pandemic has had profound impact on road accidents and driver behavior. Researchers examined the effects of the COVID-19 pandemic on driving behavior and road safety in Greece, Saudi Arabia, Cyprus, and Brazil [6]. The researchers utilized existing driving data gathered via a smartphone application and a platform developed by OSeven Telematics (https://oseven.io/). During the lockdown period, the average driving speed, speeding percentage, and harsh events (except in Cyprus) were increased.

Utilizing the same technology developed by OSeven Telematics, researchers examined the influence of COVID-19 on driving behavior and numerous safety indicators to reflect the spread of COVID-19 and the related government interventions in two nations, Greece and Saudi Arabia, during March and April 2020, when COVID-19 spread was at its peak [7]. The results showed that reduced traffic volumes due to lockdown led to a slight increase in speeds (6–11%) and an increase in harsh acceleration and harsh braking events (up to 12%). On the plus side, the number of accidents in Greece decreased by 41% during the first month of COVID-19-induced measures, and the number of people driving during the risky early morning hours (12:00–05:00 a.m.) decreased by up to 81%.

Similarly, researchers performed a quantitative assessment of the effect of COVID-19 on driving behavior during the lockdown in Greece by comparing observed values for three indicators (average speed, speeding, and harsh braking) with forecasts based on their corresponding observations prior to the lockdown [8]. The data were provided by OSeven Telematics. The results demonstrated the severity of COVID-19’s influence on driving, particularly on average speed, excessive speeding, and harsh braking per 100 km. Specifically, it was discovered that average speeds rose by 2.27 km/h compared to the forecasted evolution, while harsh braking per 100 km increased to about 1.51 on average. However, traffic accidents in Greece decreased by 49% during the COVID-19 period compared to the non-COVID-19 period. In addition, researchers identified changes in adolescent driving behavior as a result of COVID-19 restrictions [9]. It was predicted that adolescent driving would be reduced by COVID-19 restrictions. The data were based on reports provided by 58 teens in the United States over 10 weeks. Findings suggested that older, employed, minority, and adolescents with lower prosocial tendencies were less likely to limit driving activity.

Additionally, researchers intended to determine how the COVID-19 pandemic affected the chance of severe collisions in the United States by influencing driving behavior [10]. Crash data from the Virginia Department of Transportation (VDOT) were obtained, and multigroup structural equation modeling (SEM) was used to capture the complex interrelationships among crash injury severity, the context of COVID-19, driving behaviors, and other risk factors for two different groups, i.e., highways and non-highways. The results revealed that, following the emergence of COVID-19, the aggression and inattention of drivers considerably increased, resulting in a greater chance of serious collisions. In addition, researchers intended to determine whether there had been a shift in the driving style of Spanish drivers during the pandemic and to identify the group of individuals adopting a less efficient and riskier driving style [11]. The research was conducted with 30 participants, whose driving styles were compared to their driving styles before the pandemic. To do so, data from surveys and GPS were utilized. The findings indicated that drivers had adopted a more aggressive driving style than they did before the pandemic, particularly women and those who had experienced anxiety as a consequence of COVID-19.

Furthermore, researchers investigated the effect of lockdown measures used to prevent the spread of COVID-19 on road fatalities [12]. The research was based on data obtained from various online sources over a 6 month period for 15 countries (United States, Australia, Brazil, Russia, Denmark, Finland, France, Germany, Greece, Hungary, Italy, Japan, Poland, Spain, and Sweden). The 15 countries were grouped into two clusters, and the generalized linear mixed model was examined for analysis. In both clusters, there was a noticeable decline in traffic deaths. Moreover, researchers examined the impact of the stringent lockdown measures implemented in March 2020 on the driving behaviors of motorists [13]. An online survey was conducted in Canada in which 103 respondents were asked to report on their own and other drivers’ driving behaviors. After March 2020, respondents reported seeing more aggressive and distracted drivers on the road compared to before the epidemic. Moreover, researchers examined self-reported dangerous driving behaviors (speeding, distracted driving, drinking and driving, and drugged driving) in Canada and the United States during the pandemic to identify whether disparities existed between the two nations [14]. The majority of respondents claimed they did not change their behavior, whereas a small proportion reported they were less likely to participate in these dangerous driving behaviors. Nonetheless, significant proportions reported that they were more likely to engage in unsafe driving behavior during the pandemic than before it. The proportion of American drivers who claimed this was substantially greater than that of their Canadian counterparts. Furthermore, in order to evaluate the influence of the COVID-19 pandemic on driver behavior in China, researchers collected comprehensive trip data from one of the top ride-sharing firms in China from September 2019 to August 2020, including pre-, during, and post-pandemic phases in three major Chinese cities [15]. The findings revealed that, when the number of new cases grew, drivers changed the scope of their passenger search, completed fewer and shorter trips, and consequently earned less each day.

Nevertheless, there are a few key considerations about the existing studies. First, it is necessary to bear in mind that their results cannot be generalized to other places, since COVID-19 affected each country differently, as the data make evident. This seems logical, considering that the traffic restrictions enforced by governments during the COVID-19 pandemic varied from one place to another. Second, the great majority of data in previous studies were obtained through the OSeven platform or self-reports. Due to the fact that the OSeven platform follows strict information security procedures and privacy policies, all data are delivered in an anonymized manner, making it difficult to determine how individuals’ driving behaviors changed during the COVID-19 pandemic. Self-reported data have long been criticized in the scientific literature for being biased and less accurate than naturalistic driving data (NDD) [16,17,18]. Furthermore, the effect of COVID-19 on transportation can be assessed through the reports of individual academic institutions such as ETZ Zurich [19] or data companies such as Google [20], which have published summaries of COVID-19-related activity statistics. Although it is obvious from these sources that traffic drastically decreased during the lockdown period, the impact on driving behavior or road safety cannot be determined [7].

The COVID-19 pandemic in Malaysia prompted the installation of unprecedented public health measures such as requiring all residents to be restricted to a 10 km travel radius while only one person at a time from each household was permitted to travel for the purchase of necessities [21]. The impact of those lockdown measures on road safety is still not entirely known, and there are still unanswered questions, such as whether those restrictions changed drivers’ behavior in Malaysia, and, if so, which driving characteristics (speed, acceleration, braking, steering, etc.) were most influenced by those restrictions, as well as whether drivers reverted to their conduct prior to the pandemic once the traffic restrictions were lifted. To address these concerns, it is necessary to acquire NDD in order to study the effect of COVID-19 traffic restrictions on driving behavior in Malaysia to ensure the reliability of the data and findings.

This study aimed to understand the impact of the COVID-19 traffic restrictions on drivers’ behavior in Malaysia by comparing the behavior of 30 drivers before, during, and after the lockdown. In addition, the research aimed to investigate the effect the lockdown had on drivers with regard to their age and gender. Findings from this study may shed light on the effect of changing driving behaviors on safety during disruptive events such as COVID-19. Such findings will provide policymakers with a unique and informative viewpoint on how to handle road safety during future lockdowns and pandemics.

This study is organized as follows: first, a literature review on driving behavior and road safety during the pandemic is conducted, followed by an explanation of the research questions and objectives. Next is an explanation of the method used to gather driving data from different periods, followed by a description of the statistical analysis of the data. Then, the results of the changes in driving behavior during those periods are described. Afterward, the implications of COVID-19 on road safety in Malaysia are discussed and outlined. Lastly, conclusions are drawn, and recommendations for the future are provided to academics and policymakers.

## 2. Method

**Figure 1:**
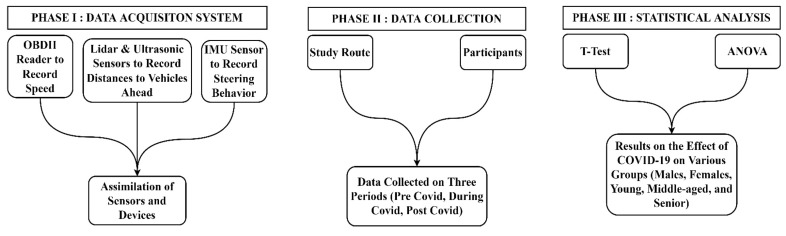
Research method.

Figure 1 shows the methodology of this research which consists of three phases.

### 2.1. Phase I: Data Acquisition System

The data acquisition system consisted of a number of sensors and devices. A reader for onboard diagnostics (OBDII) was used to record the vehicle’s speed and other driving variables, including acceleration and braking. Inside the steering wheel of the vehicle, an inertial measurement unit (IMU) was inserted to record the steering behavior of the drivers. A lidar and two ultrasonic sensors were installed in front of the vehicle in order to measure the distance between the instrumental vehicle and the vehicles in front.

**Figure 2:**
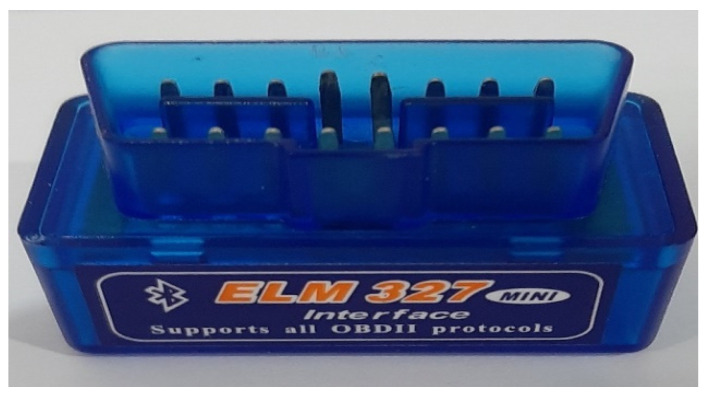
OBDII reader.

The OBDII reader sends speed, acceleration, and deceleration data in Excel format to a smartphone via Bluetooth. The OBDII reader employed in this study is shown in Figure 2.

**Figure 3:**
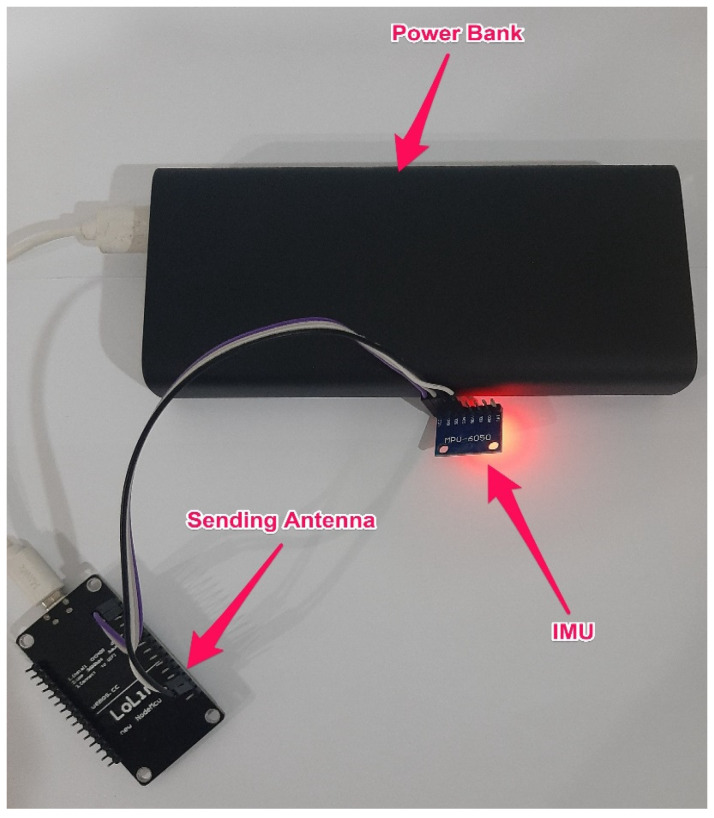
IMU, sending antenna, and power bank.

**Figure 4:**
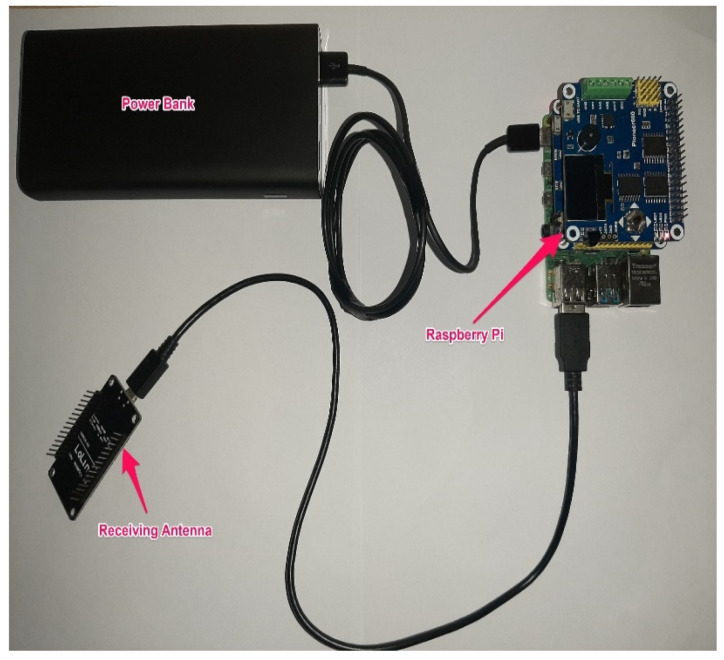
Raspberry pi, receiving antenna, and power bank.

The IMU was linked to an antenna that communicates steering data to a Raspberry Pi through a second antenna. After receiving steering data, the Raspberry Pi saves them in text file format. Figure 3 illustrates the IMU and sending antenna, as well as their respective power supplies, which were fitted inside the steering wheel. Figure 4 depicts the Raspberry Pi and receiving antenna, as well as their respective power supplies, which were placed in the trunk of the vehicle.

**Figure 5:**
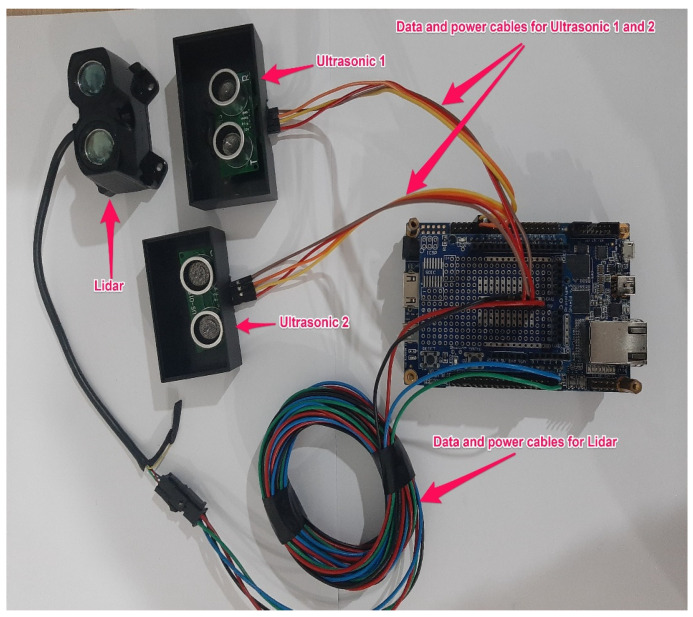
Physical connection between lidar, ultrasonic sensors, and FPGA.

The lidar and ultrasonic sensors were coupled to a field-programmable gate array (FPGA) in order to store recorded distance data in text file format. Figure 5 depicts the connection between the lidar and ultrasonic sensors (installed in front of the car) and the FPGA (installed in the trunk of the vehicle).

### 2.2. Phase II: Data Collection

**Figure 6:**
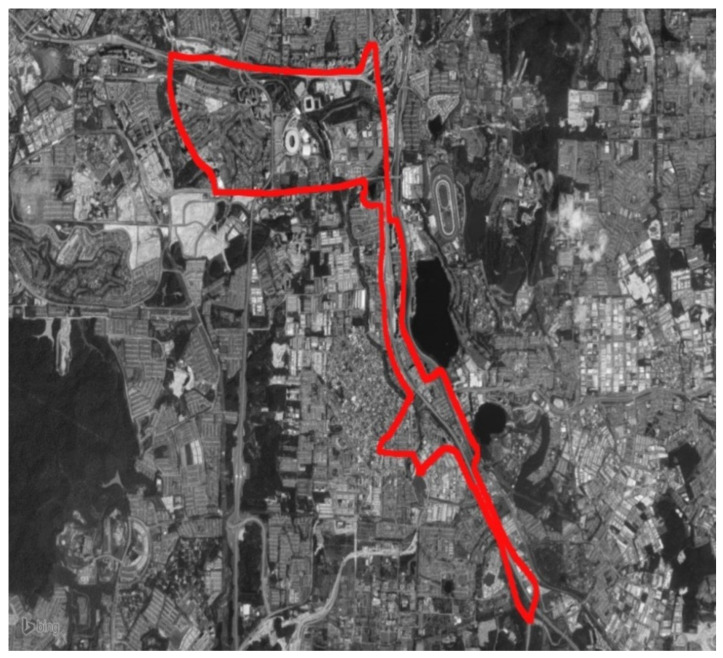
Designated route.

In contrast to the current study, which investigates the influence of COVID-19 on driving behavior using naturalistic driving data, the previous study [22] obtained driving data from 30 individuals in Malaysia to explore the effect of gender, age, cultural background, and driving day on driving behavior. This was accomplished by comparing the driving habits of younger drivers to those of middle-aged and older drivers, of male drivers to female drivers, of local Malaysian drivers to foreign drivers, and of weekday driving to weekend driving. The sample consisted of 15 males and 15 females. They varied in age from 20 to 69, and their average driving experience was 22.28 years. Figure 6 depicts the study route, which was about 25 km long and contained a variety of road types, including roundabouts and freeways, and which ran through the cities of Kuala Lumpur and Serdang. The data collection procedure was undertaken between 9:00 a.m. and 12:00 p.m. during periods of clear, sunny weather.

In this study, the same individuals were contacted to record their driving behavior during the lockdown phase. Then, they were contacted again to record their behavior after the lockdown phase. To maintain consistency and eliminate extraneous effects on the drivers, the tests were conducted at the same time (9:00 a.m. to 12:00 p.m.), on the same route, under the same weather conditions (sunny and clear), and using the same instrumented vehicle. Using a global positioning system (GPS), drivers’ whereabouts were recorded and monitored to ensure they drove on the designated route. During data gathering, the sensors of the data acquisition system captured driving data every second.

### 2.3. Phase III: Statistical Analysis

As indicated earlier, the data acquisition system captured five driving parameters for each second during data collection. These driving parameters included speed, acceleration, deceleration, steering, and distance. Researchers and experts determined that these factors were the most relevant in predicting the aggressive behavior of Malaysian drivers. The criteria for determining safe and aggressive driving in terms of speeding, distancing, acceleration, deceleration, and steering are shown in Table 1 as reported in [17,22].

Aggressive events refer to the number of times the drivers violated the safety criteria listed in Table 1. The mean and aggressive events of the five driving parameters indicated in Table 1 were used to compare the drivers’ behavior before, during, and after the implementation of traffic restrictions (lockdowns) in Malaysia. In addition, assessments of the changes in driving behavior based on gender and age during those periods were also undertaken. Consequently, participants were divided into two groups, each containing 15 individuals based on gender (males and females), and three groups, each containing 10 individuals based on age (young drivers, middle-aged drivers, and senior drivers). Analysis of variance (ANOVA) tests were conducted using the statistical package for the social sciences (SPSS) software. According to Pallant [23], significant differences were considered when the significance value was below 0.05. This rule served as the basis for the analyses in this research. Additionally, post hoc tests were used to identify specific differences between groups when ANOVA tests revealed significant differences.

## 3. Results

Table 2 displays the average and mean aggressive events in terms of the five factors (distance to vehicles ahead, speeding, acceleration, deceleration, and steering) for the 30 drivers during the three time periods.

Interestingly, no notable changes were recoded, with the exception of speeding, which increased from an average of 208 violations before COVID-19 to 216 during COVID-19;, and steering, which increased from an average of 132.52 violations before COVID-19 to 139.96 during COVID-19. Moreover, further analyses were conducted to determine whether driving behavior changed across the three time periods depending on gender and age.

The ANOVA results showed no significant differences in driving behavior across the three time periods (before, during, and after lockdown) for male drivers, as seen in Table 3. In addition, results revealed no significant differences in driving between the three periods with regard to female drivers, as seen in Table 4. Moreover, there were no significant differences in driving across the three periods with respect to senior drivers, as illustrated in Table 5. Furthermore, results indicated no significant differences in driving between the three periods for the middle-aged, as shown in Table 6. However, significant differences were found across the three periods in terms of young drivers’ average speed (sig = 0.05) and steering aggressive events (sig = 0.009), as shown in Table 7. The post hoc tests, presented in Table 8, revealed that the average speed of young drivers was substantially higher during COVID-19 traffic restrictions (mean = 51.56) than before the restrictions (mean = 46.76). Moreover, post hoc analysis revealed that young drivers performed more aggressive steering under COVID-19 traffic restrictions (mean = 160.30) than before (mean = 138.50) and after (mean = 138.30) the restrictions. The discussion section provides an in-depth explanation of the findings.

## 4. Discussion

During the lockdown period, adolescent drivers were much more aggressive in terms of steering and speeding than they were before the lockdown. This may be because young drivers found encouragement in engaging in risky driving behaviors during the lockdown period because of the low traffic volume, which impairs drivers’ ability to judge their speed adequately. This is because, when there are fewer vehicles on the road, speeding is likely to be seen as less dangerous. Such observations give insights into the influence of changed driving behaviors on safety during disruptive situations such as COVID-19.

Although previous studies examined the impact of lockdown on speeding, harsh accelerations, and rapid decelerations, the effect of lockdown on the steering behavior of drivers was not evaluated. This may be due to the fact that those studies relied mostly on surveys and observations, while this study had the benefit of using NDD to assess the effect of COVID-19 lockdown on driving behavior. Future studies in other regions are urged to assess the effect of traffic restrictions on factors such as steering and distance to vehicles ahead. Utilizing new data from other regions will aid in broadening the applicability of conclusions.

Nonetheless, some of the findings of this research are comparable to those of recent publications from other countries. In this study, there was a slight but noticeable increase in the number of aggressive events (violations) related to speeding and steering during the lockdown period compared to before the lockdown, similar to findings from a study that examined the effect of lockdown in Greece, Saudi Arabia, Cyprus, and Brazil, which revealed that speeding and harsh events (except for Cyprus) were increased during the lockdown period [6]. This increase in speed equated to 3.84%, which is relatively comparable to the results of a study conducted in Greece and Saudi Arabia, which found that lockdown caused an increase in speeding by 6–11% [7]. In Greece, average speeds increased by 2.27 km/h compared to what was predicted [8]. This study revealed that average speeds in Malaysia rose by 1.25 km/h.

In Spain, drivers have adopted more aggressive driving behavior compared to before the pandemic [11]. Moreover, data in the United States imply that driver aggression increased following the onset of COVID-19 [10], and that adolescents were less inclined to restrict their driving during the COVID-19 outbreak [9]. Such findings are corroborated by the results of this study, as aggressive behavior, particularly among young drivers, increased significantly during the lockdown compared to before it.

In a poll conducted in the United States and Canada, age was shown to have a significant influence, as older respondents were less likely to report engaging in dangerous driving behaviors during the pandemic [14]. Similarly, in this study, younger drivers showed a greater propensity for aggressive driving during the pandemic than older drivers. A Canadian study predicted that the changes in driving behaviors caused by COVID-19 would fade upon the return to more usual working situations [13]. In this study, it was shown that after the lockout, the conduct of all drivers, even young ones, returned to normal, which may support their hypothesis.

**Figure 7:**
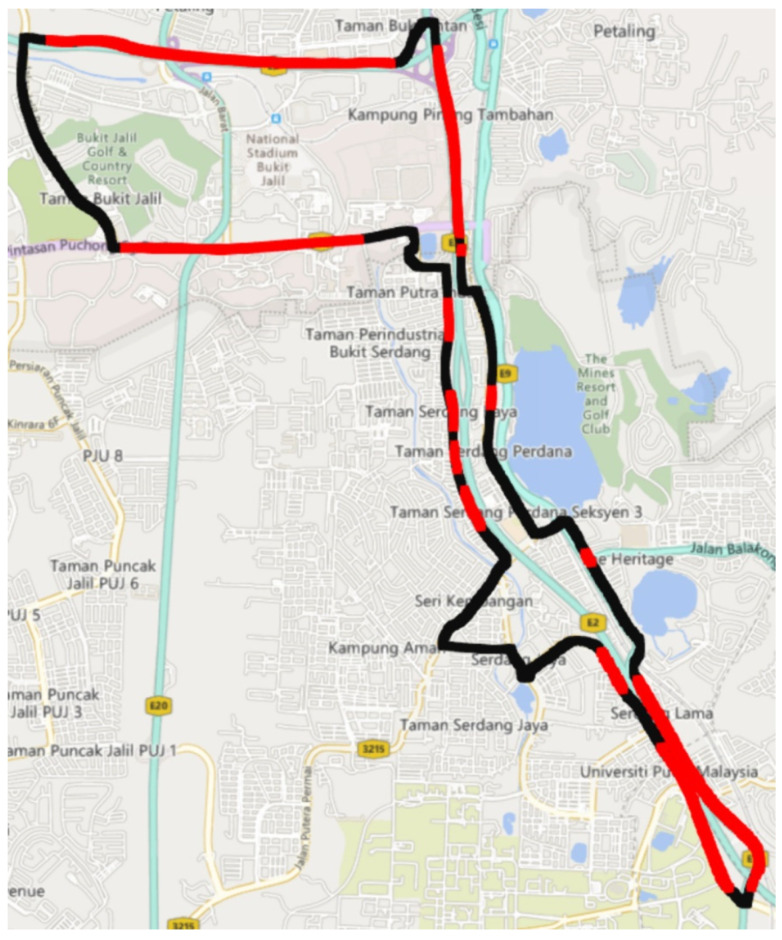
Areas where drivers exceeded speed limits.

It is worth noting that excessive speed, rapid accelerations, and rapid decelerations all contribute substantially to accidents across the globe [24]. As a result, Figure 7 shows in red the sections of the study route where drivers exceeded the legal speed limit. Policymakers should place road signs in certain places to remind drivers of the permitted speed limit and the importance of obeying stated speed limits.

When the collected data were examined, it was discovered that the most rapid accelerations occurred when the speed was between 1 and 25, and the most rapid decelerations occurred when the speed was between 33 and 72. This is due to the fact that individuals prefer to accelerate quickly after stopping at traffic signals. As a consequence, while the speed is low, rapid acceleration is not a serious worry since nearby drivers should have plenty of time to react. Rapid decelerations, on the other hand, are dangerous because neighboring vehicles may not have enough time to respond. This means that in Malaysia, rapid decelerations are more likely to cause accidents than rapid accelerations. Future researchers are urged to further investigate this topic. Nonetheless, these data show that current speed restrictions are inefficient at reducing accidents since the bulk of rapid decelerations took place within legal speed limits. Such results support the findings of [25], which concluded that aggressive driving occurs more often in low-speed zones. As a result, policymakers should change current speed limits to make rapid decelerations more restrictive in the future.

This study recommends the introduction of new speed restrictions, the installation of observational cameras, and the deployment of public awareness programs that emphasize the dangers of speeding and aggressive lane changing. Moreover, these programs should also remind people to use sanitizer every time they enter their vehicle so as not to contaminate the inside of the vehicle, disinfect their vehicle regularly, especially the dashboard and other high-touched areas, limit the number of places they drive, limit the number of passengers they transport, and ensure that anyone who shares their vehicle takes the same precautions. In response to future lockdowns, researchers, the government (police, transportation ministry, and health ministry), and the business sector (insurance companies) should work together to gather traffic data and develop road safety programs.

## 5. Conclusions

This study expands on a previous study in which the authors developed an acquisition system and acquired driving data from 30 participants prior to the COVID-19 lockdown in Malaysia. Driving data were obtained from the same individuals twice in this study: during COVID-19 lockdown in 2020 and after COVID-19 lockdown in 2022. Speeding, harsh acceleration, harsh deceleration, close distancing to vehicles ahead, and sharp steering were identified as factors for aggressive driving, and the mean and aggressive events of those factors were used to compare drivers’ behavior before, during, and after the implementation of lockdowns in Malaysia. In addition, assessments of changes in driving behavior with regard to gender and age were conducted during those periods. As a result, participants were categorized into two gender groups (males and females) and three age groups (young drivers, middle-aged drivers, and senior drivers). The results revealed that, during the lockdown period, drivers were more likely to speed and steer aggressively than before and after the lockdown period. However, ANOVA tests revealed no significant differences in driving behavior among male and female drivers over the three time periods (before, during, and after lockdown). Furthermore, no significant differences in driving behavior were observed among middle-aged and senior drivers during those periods. However, ANOVA tests revealed significant differences in driving behavior among young drivers during the lockdown period compared to before and after the lockdown period in terms of speeding and aggressive steering. These findings were comparable to studies conducted in other nations. Moreover, the research highlights the areas of the study route where vehicles exceeded the legal speed limit in order to assist policymakers in improving traffic safety in those regions. Furthermore, the research investigated how most rapid accelerations and decelerations occurred within acceptable speed limits and provided guidance on how to modify existing speed limits to curb such aberrant behaviors. The findings provide a unique and valuable insight into how drivers’ behavior changed during the pandemic and will help guide future studies into investigating unsafe driving behavior during further COVID-19 lockdowns and future pandemics. The findings imply that, during the current epidemic and any future resurgences of COVID-19 or other pandemics, tailored regulatory measures should especially target speeding and lane changes to help reduce aggressive driving behavior.

The applicability of the study’s findings could be limited to countries with comparable COVID-19 mitigation efforts and restrictions. Therefore, in the future, researchers should concentrate on assessing the effects of COVID-19 in other countries in order to compare and evaluate various impacts on driving behavior. Moreover, additional driving behaviors, such as cell phone usage while driving and being distracted driving, might aid in assessing the impacts of lockdown on driving. Furthermore, in the post-COVID era, researchers should investigate how tourism may have a detrimental impact on traffic safety in high-traffic areas, particularly after such a lengthy lockdown period.

This study contributes to the greater good of society by providing policymakers with guidelines and recommendations for enhancing traffic safety in an effort to save the lives of pedestrians and other road users, as well as by providing scientific knowledge regarding the effects of global pandemics on factors other than health and economy, and by encouraging researchers in other regions to conduct comparative studies.

## Figures and Tables

**Table 1:** Criteria for identifying safe and aggressive behaviors.

Parameter	Criteria	Status
Speed	≤speed limit	Safe
	>speed limit	Aggressive
Distance to vehicle ahead	≥4 m for every 15 km/h	Safe
	<4 m for every 15 km/h	Aggressive
Acceleration	<3.5 m/s^2^	Safe
	≥3.5 m/s^2^	Aggressive
Deceleration	>−5.5 m/s^2^	Safe
	≤−5.5 m/s^2^	Aggressive
Steering	If *z*-score for the change in yaw angle per second is between 1σ and −1σ	Safe
	If *z*-score for the change in yaw angle per second is above 1σ or below −1σ	Aggressive

**Table 2:** Average and mean aggressive events for the five factors during the three periods.

	Distance to Vehicle Ahead	Speed	Acceleration	Deceleration	Steering
**Pre-COVID**	A = 1107.09 cm MAE = 385.86	A = 45.41 kmph MAE = 208.93	A= 0.64 m/s^2^ MAE = 0.53	A = −0.78 m/s^2^ MAE = 0.26	A = 9.31 MAE = 132.53
**During COVID**	A = 1107.05 cm MAE = 386.06	A = 46.66 kmph MAE = 216.73	A= 0.64 m/s^2^ MAE = 0.60	A = −0.78 m/s^2^ MAE = 0.30	A = 9.28 MAE = 139.96
**Post-COVID**	A = 1107.05 cm MAE = 386.33	A = 45.26 kmph MAE = 209.20	A= 0.65 m/s^2^ MAE = 0.53	A = −0.79 m/s^2^ MAE = 0.26	A = 9.30 MAE = 132.33
A = average MAE = mean aggressive events					

**Table 3:** Statistical differences for male drivers.

Variable	Group	*N*	Mean	F	Sig.	Conclusion
Distance to vehicle ahead	Pre-COVID	15	1067.16	0	1.0	No differences.
(Mean)	During COVID	15	1066.4			
	Post-COVID	15	1066.46			
	Total	45	1066.67			
Speed	Pre-COVID	15	42.6	0.002	0.998	No differences.
(Mean)	During COVID	15	42.6			
	Post-COVID	15	42.5			
	Total	45	42.6			
Acceleration	Pre-COVID	15	0.65	0.253	0.778	No differences.
(Mean)	During COVID	15	0.64			
	Post-COVID	15	0.66			
	Total	45	0.65			
Deceleration	Pre-COVID	15	−0.767	0.101	0.904	No differences.
(Mean)	During COVID	15	−0.733			
	Post-COVID	15	−0.733			
	Total	45	−0.771			
Steering	Pre-COVID	15	9.187	0.001	0.999	No differences.
(Mean)	During COVID	15	9.180			
	Post-COVID	15	9.193			
	Total	45	9.187			
Distance to vehicle ahead	Pre-COVID	15	458.8	0	1.0	No differences.
(Aggressive events)	During COVID	15	458.6			
	Post-COVID	15	458.9			
	Total	45	458.8			
Speed	Pre-COVID	15	187.67	0.026	0.974	No differences.
(Aggressive events)	During COVID	15	192.87			
	Post-COVID	15	188.13			
	Total	45	189.56			
Acceleration	Pre-COVID	15	0.4	0	1.0	No differences.
(Aggressive events)	During COVID	15	0.4			
	Post-COVID	15	0.4			
	Total	45	0.4			
Deceleration	Pre-COVID	15	0.27	0	1	No differences.
(Aggressive events)	During COVID	15	0.27			
	Post-COVID	15	0.27			
	Total	45	0.27			
Steering	Pre-COVID	15	141.8	0.131	0.878	No differences.
(Aggressive events)	During COVID	15	145.6			
	Post-COVID	15	141.33			
	Total	45	142.91			

**Table 4:** Statistical differences for female drivers.

Variable	Group	*N*	Mean	F	Sig.	Conclusion
Distance to vehicle ahead	Pre-COVID	15	1147.027	0	1.0	No differences.
(Mean)	During COVID	15	1147.700			
	Post-COVID	15	1147.647			
	Total	45	1147.458			
Speed	Pre-COVID	15	48.27	1.469	0.242	No differences.
(Mean)	During COVID	15	50.680			
	Post-COVID	15	47.980			
	Total	45	48.956			
Acceleration	Pre-COVID	15	0.633	0.091	0.913	No differences.
(Mean)	During COVID	15	0.633			
	Post-COVID	15	0.640			
	Total	45	0.636			
Deceleration	Pre-COVID	15	−0.793	0.333	0.718	No differences.
(Mean)	During COVID	15	−0.787			
	Post-COVID	15	−0.807			
	Total	45	−0.796			
Steering	Pre-COVID	15	9.440	0.016	0.984	No differences.
(Mean)	During COVID	15	9.387			
	Post-COVID	15	9.420			
	Total	45	9.416			
Distance to vehicle ahead	Pre-COVID	15	312.87	1.055	1.0	No differences.
(Aggressive events)	During COVID	15	313.53			
	Post-COVID	15	313.73			
	Total	45	313.38			
Speed	Pre-COVID	15	230.20	0.084	0.920	No differences.
(Aggressive events)	During COVID	15	240.60			
	Post-COVID	15	230.27			
	Total	45	233.69			
Acceleration	Pre-COVID	15	0.67	0.096	0.909	No differences.
(Aggressive events)	During COVID	15	0.8			
	Post-COVID	15	0.87			
	Total	45	0.71			
Deceleration	Pre-COVID	15	0.27	0.034	0.966	No differences.
(Aggressive events)	During COVID	15	0.33			
	Post-COVID	15	0.27			
	Total	45	0.29			
Steering	Pre-COVID	15	123.27	0.844	0.437	No differences.
(Aggressive events)	During COVID	15	134.33			
	Post-COVID	15	123.33			
	Total	45	126.98			

**Table 5:** Statistical differences for senior drivers.

Variable	Group	*N*	Mean	F	Sig.	Conclusion
Distance to vehicle ahead	Pre-COVID	10	980.78	0	1.0	No differences.
(Mean)	During COVID	10	980.22			
	Post-COVID	10	980.03			
	Total	30	980.34			
Speed	Pre-COVID	10	41.99	0.002	0.998	No differences.
(Mean)	During COVID	10	41.37			
	Post-COVID	10	41.94			
	Total	30	41.76			
Acceleration	Pre-COVID	10	0.64	0.253	0.778	No differences.
(Mean)	During COVID	10	0.64			
	Post-COVID	10	0.65			
	Total	30	0.64			
Deceleration	Pre-COVID	10	−0.76	0.071	0.931	No differences.
(Mean)	During COVID	10	−0.77			
	Post-COVID	10	−0.77			
	Total	30	−0.76			
Steering	Pre-COVID	10	8.90	0.013	0.987	No differences.
(Mean)	During COVID	10	8.90			
	Post-COVID	10	8.87			
	Total	30	8.89			
Distance to vehicle ahead	Pre-COVID	10	502.50	0	1.0	No differences.
(Aggressive events)	During COVID	10	502.30			
	Post-COVID	10	502.20			
	Total	30	502.33			
Speed	Pre-COVID	10	208.20	0.026	0.974	No differences.
(Aggressive events)	During COVID	10	209.30			
	Post-COVID	10	208.50			
	Total	30	208.67			
Acceleration	Pre-COVID	10	0.3	0	1.0	No differences.
(Aggressive events)	During COVID	10	0.3			
	Post-COVID	10	0.3			
	Total	30	0.3			
Deceleration	Pre-COVID	10	0.10	0	1	No differences.
(Aggressive events)	During COVID	10	0.10			
	Post-COVID	10	0.10			
	Total	30	0.10			
Steering	Pre-COVID	10	138.10	0.001	0.999	No differences.
(Aggressive events)	During COVID	10	138.10			
	Post-COVID	10	137.60			
	Total	30	137.93			

**Table 6:** Statistical differences for middle-aged drivers.

Variable	Group	*N*	Mean	F	Sig.	Conclusion
Distance to vehicle ahead	Pre-COVID	10	1194.85	0	1.0	No differences.
(Mean)	During COVID	10	1194.73			
	Post-COVID	10	1194.58			
	Total	30	1194.72			
Speed	Pre-COVID	10	47.49	0.025	0.976	No differences.
(Mean)	During COVID	10	47.06			
	Post-COVID	10	47.27			
	Total	30	47.27			
Acceleration	Pre-COVID	10	0.65	0.122	0.886	No differences.
(Mean)	During COVID	10	0.64			
	Post-COVID	10	0.65			
	Total	30	0.647			
Deceleration	Pre-COVID	10	−0.80	0.466	0.633	No differences.
(Mean)	During COVID	10	−0.79			
	Post-COVID	10	−0.81			
	Total	30	−0.80			
Steering	Pre-COVID	10	9.25	0.035	0.966	No differences.
(Mean)	During COVID	10	9.18			
	Post-COVID	10	9.27			
	Total	30	9.23			
Distance to vehicle ahead	Pre-COVID	10	367.60	0	1.0	No differences.
(Aggressive events)	During COVID	10	368.00			
	Post-COVID	10	368.70			
	Total	30	368.10			
Speed	Pre-COVID	10	182.70	0	1.0	No differences.
(Aggressive events)	During COVID	10	183.40			
	Post-COVID	10	182.80			
	Total	30	182.97			
Acceleration	Pre-COVID	10	0.4	0	1.0	No differences.
(Aggressive events)	During COVID	10	0.4			
	Post-COVID	10	0.4			
	Total	30	0.4			
Deceleration	Pre-COVID	10	0.10	0	1	No differences.
(Aggressive events)	During COVID	10	0.10			
	Post-COVID	10	0.10			
	Total	30	0.10			
Steering	Pre-COVID	10	121.00	0.131	0.878	No differences.
(Aggressive events)	During COVID	10	121.50			
	Post-COVID	10	121.10			
	Total	30	121.20			

**Table 7:** Statistical differences for young drivers.

Variable	Group	*N*	Mean	F	Sig.	Conclusion
Distance to vehicle ahead	Pre-COVID	10	1145.65	0	1.0	No differences.
(Mean)	During COVID	10	1146.20			
	Post-COVID	10	1145.55			
	Total	30	1146.13			
Speed	Pre-COVID	10	46.76	3.298	0.05	Significant differences with relation to average speed.
(Mean)	During COVID	10	51.56			
	Post-COVID	10	46.57			
	Total	30	48.29			
Acceleration	Pre-COVID	10	0.64	0.123	0.885	No differences.
(Mean)	During COVID	10	0.64			
	Post-COVID	10	0.65			
	Total	30	0.64			
Deceleration	Pre-COVID	10	−0.78	0.089	0.915	No differences.
(Mean)	During COVID	10	−0.78			
	Post-COVID	10	−0.79			
	Total	30	−0.78			
Steering	Pre-COVID	10	9.79	0.001	0.999	No differences.
(Mean)	During COVID	10	9.77			
	Post-COVID	10	9.78			
	Total	30	9.78			
Distance to vehicle ahead	Pre-COVID	10	287.50	0	1.0	No differences.
(Aggressive events)	During COVID	10	287.90			
	Post-COVID	10	288.10			
	Total	30	287.83			
Speed	Pre-COVID	10	235.9	0.286	0.754	No differences.
(Aggressive events)	During COVID	10	257.50			
	Post-COVID	10	236.30			
	Total	30	243.23			
Acceleration	Pre-COVID	10	0.90	0.117	0.890	No differences.
(Aggressive events)	During COVID	10	1.10			
	Post-COVID	10	0.90			
	Total	30	0.97			
Deceleration	Pre-COVID	10	0.60	0.029	0.971	No differences.
(Aggressive events)	During COVID	10	0.70			
	Post-COVID	10	0.60			
	Total	30	0.63			
Steering	Pre-COVID	10	138.50	5.578	0.009	Significant differences with relation to steering.
(Aggressive events)	During COVID	10	160.30			
	Post-COVID	10	138.30			
	Total	30	145.70			

**Table 8:** Post hoc results to highlight differences between periods for young drivers.

Dependent Variable	(I)	(J)	Sig.	Comment
Speed	Pre-COVID	During COVID	0.093	Young drivers drove more slowly before COVID.
(Mean)		Post-COVID	0.996	
	During COVID	Pre-COVID	0.093	Young drivers drove more quickly during COVID.
		Post-COVID	0.078	
	Post-COVID	Pre-COVID	0.996	No differences.
		During COVID	0.078	
Steering	Pre-COVID	During COVID	0.020	Young drivers performed less aggressive steering before COVID.
(Aggressive events)		Post-COVID	1.0	
	During COVID	Pre-COVID	0.020	Young drivers performed more aggressive steering during COVID.
		Post-COVID	0.019	
	Post-COVID	Pre-COVID	1.0	Young drivers performed less aggressive steering after COVID.
		During COVID	0.019	

## Data Availability

Data can be provided by the authors upon request.
